# Genetic variation of *HvXYN1* associated with endoxylanase activity and TAX content in barley (*Hordeum vulgare* L.)

**DOI:** 10.1186/s12870-019-1747-5

**Published:** 2019-04-30

**Authors:** Xueli Lu, Yunxia Fang, Bin Tian, Tao Tong, Jiahui Wang, Hua Wang, Shengguan Cai, Jiang Hu, Dali Zeng, Heng Xu, Xiaoqin Zhang, Dawei Xue

**Affiliations:** 1 College of Life and Environmental Sciences, Hangzhou Normal University, 16 Xiasha Road, Hangzhou, 310036 China; 2 State Key Laboratory Breeding Base for Zhejiang Sustainable Pest and Disease Control, Zhejiang Academy of Agricultural Science, 298 Deshengzhong Road, Hangzhou, 310021 China; 3 Agronomy Department, Key Laboratory of Crop Germplasm Resource of Zhejiang Province, Zhejiang University, 866 Yuhangtang Road, Hangzhou, 310058 China; 4 State Key Laboratory of Rice Biology, China National Rice Research Institute, 359 Tiyu Road, Hangzhou, 310006 China

**Keywords:** *HvXYN1*, Arabinoxylan, Endo-β-1,4-xylanase, Single nucleotide polymorphism, Beer barley

## Abstract

**Background:**

Endo-β-1,4-xylanase1 (EA), the key endoxylanase in plants, is involved in the degradation of arabinoxylan during grain germination. In barley (*Hordeum vulgare* L.), one gene (*HvXYN-1*) that encode a endo-beta-1,4-xylanase, has been cloned. However, the single nucleotide polymorphisms (SNPs) that affect the endoxylanase activity and total arabinoxylan (TAX) content have yet to be characterized. The investigation of genetic variation in *HvXYN1* may facilitate a better understanding of the relationship between TAX content and EA activity in barley.

**Results:**

In the current study, 56 polymorphisms were detected in *HvXYN1* among 210 barley accessions collected from 34 countries, with 10 distinct haplotypes identified. The SNPs at positions 110, 305, 1045, 1417, 1504, 1597, 1880 bp in the genomic region of *HvXYN1* were significantly associated with EA activity (*P* < 0.0001), and the sites 110, 305, and 1045 were highly significantly associated with TAX content. The amount of phenotypic variation in a given trait explained by each associated polymorphism ranged from 6.96 to 9.85%. Most notably, we found two variants at positions 1504 bp and 1880 bp in the second exon that significantly (*P* < 0.0001) affected EA activity; this result could be used in breeding programs to improve beer quality. In addition, African accessions had the highest EA activity and TAX content, and the richest germplasm resources were from Asia, indicating the high potential value of Asian barley.

**Conclusion:**

This study provided insight into understanding the relationship, EA activity, TAX content with the SNPs of *HvXYN1* in barley. These SNPs can be applied as DNA markers in breeding programs to improve the quality of barley for beer brewing after further validation.

**Electronic supplementary material:**

The online version of this article (10.1186/s12870-019-1747-5) contains supplementary material, which is available to authorized users.

## Background

Barley (*Hordeum vulgare* L.) is an ancient crop that is distributed worldwide. Barley is an important raw material in beer brewing [1], and its quality directly affects the fermentation ability, flavor, turbidity or foam stability of beer. Studies of the molecular mechanisms underlying genetic and environmental variation and the quality differences in grain protein content, β-glucan content, β-amylase, limit dextrin enzyme activity and other traits related to barley quality have been reported [2–9]. Arabinoxylan (AX) is the principal non-cellulosic polysaccharide in the thick aleurone layer of the barley grain cell wall [10]. AX has an important influence on the brewing quality of barley, as it can affect the grain hardness and water absorption of seeds, hindering the release of endosperm substances. In the beer-brewing process, insufficient degradation of AX will hinder the release of hydrolytic enzymes from the thick aleurone layer or nutrients from the endosperm, and almost the non-degradable AX would flow into wort, increase viscosity, block subsequent filtration and processing, increase the turbidity of beer and influenced the flavor of beer or other beer qualities [11–13].

AX, which consists of a linear 1,4-linked backbone of D-xylopyranosyl residues with various side chains, is the main component of hemicellulose in cereals [14]. These side chains in AXs have been found to increase wort viscosity and decrease filterability [11, 12].. AXs are mainly degraded by the glycosyl hydrolases endo-β-1,4-xylanase (EA, further referred to as xylanase) (EC3.2.1.8), which can hydrolyse β-1,4-linkages between xylose residues in the backbone of these polysaccharides, and exo-β-1,4-xylosidase (EC3.2.1.37) [15, 16]. In cereals, these enzymes are involved in the depolymerization of arabinoxylan during seed germination [17]. Particularly in barley, xylanase can be synthesized and secreted in the aleurone layer [18]. However, due to the activity of xylanase in malt, a large amount of arabinoxylan is dissolved [13, 19], which in turn improves the viscosity, foam stability and sensory characteristics of beer [20].

Three endo-β-1,4-xylanase isoenzymes have been purified from germinating barley and shown to be endo-hydrolases on the basis of product analysis [21]. A cDNA which encoding endo-β-1,4-xylanase,with molecular weight of 41,000 D (Mr41 000) has been characterized during germinating barley [22, 23]. Caspers et al. [24] have identified the other major endo-β-1,4-xylanase (*XYN-1*) in the aleurone of germinating barley grains and the gene located in the long arm of chromosome 5H. It is expressed as a precursor of molecular weight 61,500 D (Mr61 500) with both N- and C-terminal pro-peptides and regulated the disintegration of aleurone cell.

The ability of different crop species to meet people’s needs is the result of sequence variation in genes in the accessions of the given crop species. Single nucleotide polymorphisms (SNPs) and small insertions and deletions (indels) are the most common forms of genetic variation in natural crop populations, and these polymorphisms may reflect the relationship of phenotypic variation and plant adaptation in different environments, thus playing a prominent role in the heritability of phenotypes [25]. Recently, association analysis has emerged as a powerful approach to identify the role of genetic polymorphisms in the phenotypic variation in beer barley. For example, Mohsen et al. [26] collected 1862 barley breeding lines and evaluated in 97 field trials about malting quality traits in barley through genome-wide association study. They found 108 and 107 significant marker-trait associations malting quality in all six-row and all two-row breeding programs and the distribution of favorable alleles for marker-assisted selection and germplasm exchange. Null LOX-1 activity varieties with stable foam in beer and better flavor of beer always were screened out as malting barley. Guo et al. [20] identified a rare C/G mutation (SNP-61) in the second intron which result in null LOX-1 activity through an altered splicing acceptor site and the SNP could be used in breeding programs for barley to be used for malting. In general, superior malting quality requires a high conversion of starch to fermentable sugars. Gong et al. [27] selected one gene, encodingβ-amylase, which could convert starch to sugars during malting as an indicator to improve malting quality; their results demonstrated that the broad variation in Bmy1 could provide novel alleles for the improvement of diastatic power and malting quality. Jin et al. [28] used structure-based association analysis to demonstrate that the key SNPs of HvLDI associated with limit dextrinase (LD) activity could be used to increase the conversion efficiency of conversion of starch to sugars during malting and improve the quality of beer. Hassan et al. [29] revealed three significant quantitative trait loci (QTL) associated with grain AX levels in barley through genome-wide association analysis., while they did not mentione the gene *HvXYN1* The research conclusions concerning AX content are quite clear, but there are few studies of the relationship of EA activity and TAX content. To date, allelic variation of *HvXYN1* in barley has not been systematically examined. Exploration of genetic variation in *HvXYN1* may provide a better understanding of the functions of *HvXYN1* and may yield useful information for improving the quality of barley.

In barley, only Caspers et al. [24] have cloned the endo-beta-1,4-xylanase gene (*XYN-1*)*.* However, the key SNPs that were related to endo-xylanase activity and xylan content have yet to be reported. Therefore, to evaluate nucleotide diversity and to perform neutrality tests of detected regions, we detected allelic variation in the targeted region of *HvXYN1* in 210 cultivated barley micro-nucleus germplasms collected from diverse geographical areas. In addition, association analysis between the allelic variations and EA activity and TAX content was performed to identify the key SNPs that significantly influencing TAX content and EA activity indifferent barley grain and to provide guidance for developing an allele-specific marker and improving beer barley quality by these key SNPs.

## Results

### Phenotypic variation

**Fig. 1:**
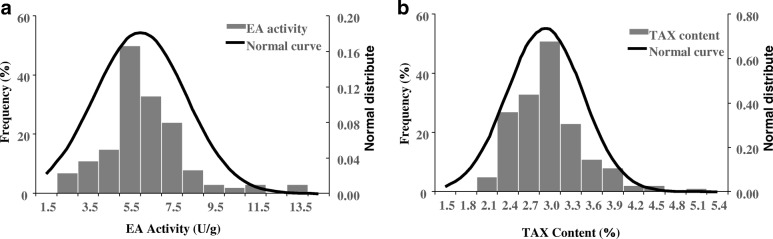
The frequency distribution of EA activity and TAX content of the examined barley accessions. The histogram indicates the phenotypic distribution frequency, and the curve indicates the fitted normal curve. The X axis represents EA activity and TAX content; the Y axis represents frequency of EA activity and TAX content. **a** The frequency distribution of EA activity. **b** The frequency distribution of TAX content

Significant amounts of genetic variation in EA activity and TAX content were observed among the 210 barley accessions (Fig. 1). The mean value of EA activity was 2.95 U/g, ranging from 1.97 U/g to 5.08 U/g; the value for TAX content was 5.95%, ranging from 1.76 to 13.29%. Moreover, normal distributions of EA activity and TAX content were observed (Fig. 1a, b), suggesting that multiple genes/QTLs control the focal traits in barley. Large phenotypic variation was observed for all traits; on the whole, the mean coefficient of variation (CV value) of TAX content (36%) was much larger than that of EA activity (19%), suggesting that the greater variation in TAX content is mainly controlled by genetic factors and is affected by environmental variation. In addition, the TAX content variation reached 36%, which further indicated that it was more vulnerable to environmental variation. The values of the two traits were significantly and positively correlated (R^2^ = 0.135*) (Table 1).

**Table 1:** Phenotypic scores of EA activity and TAX content for 210 barley accessions

Trait	N	Mean ± SD	CV (%)	Range	skewness	kurtosis	R^2^ Spearman TAX
TAX (%)	210	5.95 ± 2.16	36	1.765 ± 13.297	1.065	1.328	–
EA (U/g)	210	2.94 ± 0.56	19	1.97 ± 5.085	0.955	1.542	0.135*

Note: The first column indicates the main traits of xylan. *Correlation (R^2^) of TAX and EA is significant at the 0.05 level (2 tailed). *EA* Endo-β-1,4-xylanase activity, *TAX* Total arabinoxylan content, *N* number of barley accessions tested, *SD* standard deviation.*Correlation is significant at the 0.05 level (2-tailed)

### Nucleotide polymorphisms in *HvXYN1*

By amplifying the genomic region of *HvXYN1* which is located in the long arm of chromosome 5H in barley, 142 natural variation sites were identified among the 210 accessions by sequencing analysis (Table 2). Nucleotide diversity (π) of the targeted region of *HvXYN1* was 0.00736 across 210 barley accessions. The π values differed among geographic regions, ranging from 0.0018 for east European accessions (4 accessions) to 0.0103 for east Asia accessions (46 accessions) (Table 2). To test whether the identified nucleotide polymorphisms were selectively neutral, D*, F* and Tajima’s D statistics were calculated. For the 210 barley accessions, Tajima’s D value was − 1.48, but this was not significant (*P* > 0.05). However, the values of D* and F* were highly significant. Apart from the Asian and European accessions, other subgroups were from Africa, Middle East Asia and North East Asia, suggesting that the gene has not been under strong selection. However, the values of Tajima’s D from East Africa were statistically significant (*P* < 0.05) (Table 2). This suggests that nucleotide variation in the *HvXYN1* gene in Africa did not result from standard neutral evolution.

**Table 2:** Barley *HvXYN1* nucleotide diversity (π), haplotype diversity and selection (D* and F*, and Tajima’s D) statistics for each geographic region

Population	Number of accessions	Number of polymorphic sites (π)	Nucleotide diversity	Number of haplotypes	Haplotpe diversity	D*	F*	Tajima’s D
Total	210	142	0.00736	80	0.923	4.642**	−3.733**	−1.484
EAF	30	19	0.00438	4	0.603	1.290*	1.823*	2.124*
NAF	7	32	0.00709	5	0.806	0.758	0.825	0.666
ASI	112	141	0.00903	60	0.954	−3.522**	−3.133**	−1.426
WAS	47	60	0.00709	23	0.909	−1.245	−1.034	−0.194
EAS	47	123	0.0103	30	0.952	−1.735	−1.830	− 1.186
SAS	16	53	0.00901	11	0.943	−0.051	−0.029	0.0416
WEU	9	21	0.00477	5	0.833	0.306	0.461	0.742
CEU	19	36	0.00573	10	0.915	−0.138	−0.102	0.032
EEU	5	8	0.0018	3	0.700	−0.807	− 0.845	−0.807
SEU	17	30	0.00473	6	0.743	−0.099	−0.130	− 0.145
EUR	61	39	0.00492	23	0.855	0.367	0.350	0.163

Note: *AFR* Africa, *EUR* Europe, *ASI* Asia; *EAF* East Africa, *NAF* North Africa, *EAS* East Asia, *WAS* West Asia, *SAS* South Asia, *WEU* West Europe, *CEU* Central Europe, *EEU* East Europe, *SEU* South Europe, *, *P* < 0.05; **, *P* < 0.01

### Population structure and genetic diversity

**Fig. 2:**
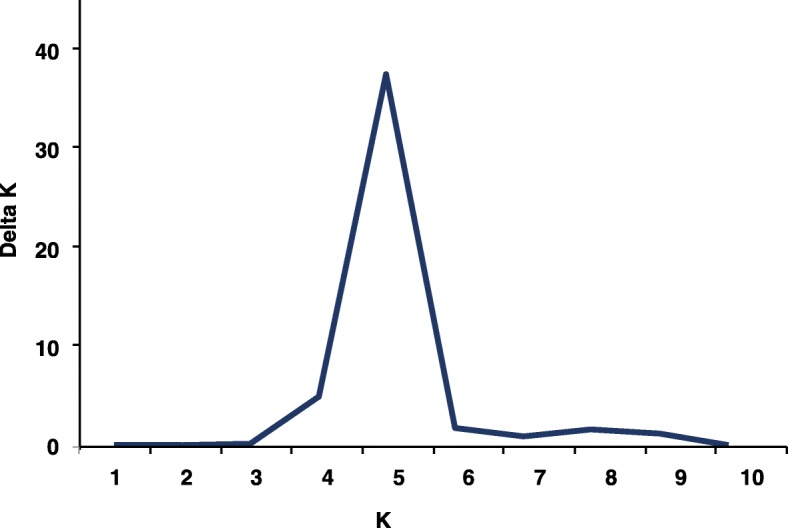
Estimation of the most probable number of clusters k, based on 10 independent runs and k ranging from1 to10. The delta K value reached a peak at K = 5, indicates relatively low levels of the admixture with five subgroups in the panel. The X axis represents K, and the Y axis represents delta K

**Fig. 3:**
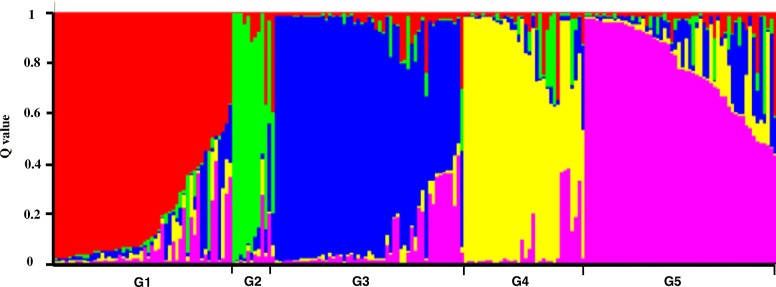
Population structure of 210 barley accessions based on 35 SSRs (K = 5). Five colors indicate the subpopulations G1, G2, G3, G4 and G5, respectively. Each accession is represented by a thin vertical line with the lengths proportional to each of the subpopulations. The y-axis is the subgroup membership, and the x-axis is the accessions in the five groups (G1, G2, G3, G4 and G5)

In the correlation analysis, the population structure was taken into account to avoid false positive results. Analysis of genetic distance and population structure confirmed the presence of significant structure in the barley population. The 35 SSR markers were used to evaluate the subset of 210 varieties genotypes. Stratification within the barley population was detected by STRUCTURE. The largest value of the statistical index ΔK was used as an indicator of the most probable number of subpopulations for all accessions (Fig. 2). The ΔK value attained a clear maximum at K = 5, and the five groups (or clusters) revealed relatively low levels of admixture, with groups G1, G2, G3, G4 and G5 containing 46,13,47,43 and 51 accessions, respectively (Fig. 3).

### Species geographical information

**Fig. 4:**
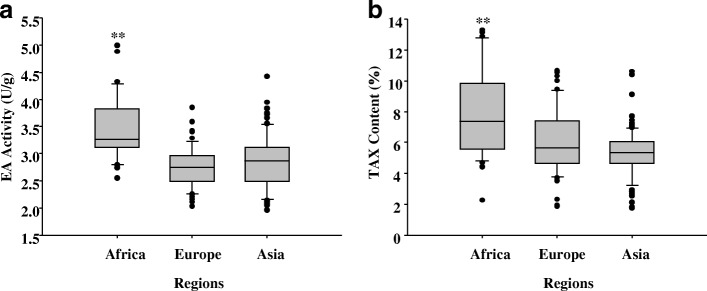
Comparisons of the EA activity and TAX content in different regions. **a** EA activity contrasts in different geographic regions. **b** TAX content contrasts in different geographic regions. ** indicates highly significant (*P* < 0.01) difference between means. The Duncan multiple range test and critical test were conducted if the analyses were significant (*P* < 0.05)

The geographical distribution of the 210 cultivars used in the analysis comprised 37 from Africa, 112 from Asia, and 61 from Europe (Table 3). Analysis of regional information of EA activity and TAX content revealed that EA activity in cultivars from Africa (3.487 U/g) was significantly higher than that of cultivars from Asia and Europe. In addition, Europe (2.781 U/g) had the lowest activity (Fig. 4a and Additional file 1: Table S1); African cultivars had significantly higher TAX content than those from Asia and Europe, with Asia being the lowest (Fig. 4b and Additional file 1: Table S1).

**Table 3:** Geographic origins of the barley accessions used in the study

Continent	Geographical region	Country
Africa (37)	Northern (7)	Morocco (1), Tunisia (2), Egypt (4)
	Eastern (30)	Ethiopia (30)
Asia (112)	Western (47)	Syria (4), Iran (7), Armenia (3), Georgia (2), Azerbaijan (3), Iraq (12), Turkey (16)
	Middle (2)	Afghanistan(2)
	Eastern (47)	Korea (20), China (13), Japan (14)
	Southern (16)	Pakistan (1), India (3), Nepal (12)
Europe (61)	Western (13)	France (5), United Kingdom (4)
	Central (19)	Germany (4), Switzerland (3), Czechoslovakia (7), Poland (4)
	Eastern (5)	Bulgaria (4), Union of Soviet Socialist Republics (1)
	Southern (17)	Italy (4), Yugoslavia (3), Rumania (5), Hungary (1), Spain (4)
	Northern (7)	Finland (3), Denmark (2), Sweden (2)

Note: Numbers in brackets indicate how many accessions from each country

### Associated SNP loci and haplotypes

**Fig. 5:**
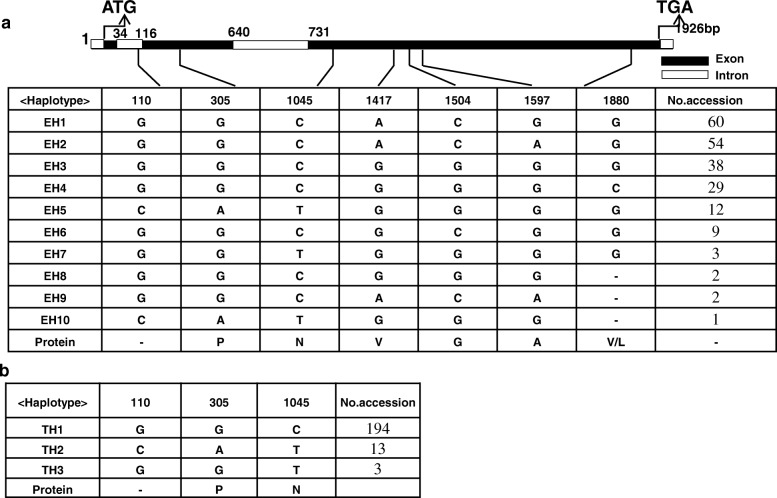
Haplotype analysis of the *HvXYN1* gene region in the 210 accessions of EA activity and TAX content. The *XYN1* sequence contained three exons (indicated in black boxes), two introns (indicated in white boxes) and the full length of 1926 bp genome is shown at the top of the diagram. The position of each SNP is shown in the first row as described in Table 4 (SNP frequency > 5%). Ten haplotypes (EH1-EH10) were detected in the EA activity (**a**) and three were detected in the TAX activity (**b**) among all the accessions. The number of accessions (No. accession) in different haplotypes is shown in the far right columns, and amino acid change variation corresponding to the SNPs is shown in the last row. Capital letters in this row represent the amino acid as follows P: Proline; N: Asparagine; V: Valine; G: Glycine; A: Alanine; V: Valine; L: Leucine

**Fig. 6:**
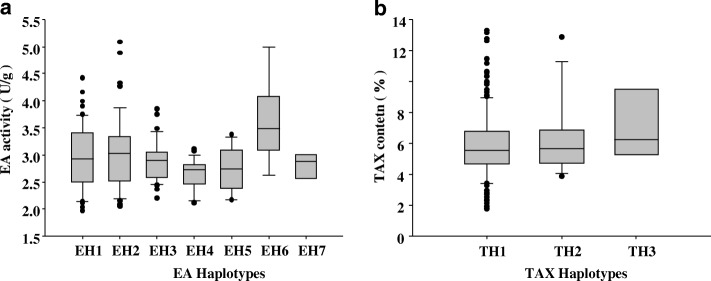
EA activity and TAX content contrasts in different haplotypes. **a** EA activity contrasts in different haplotypes (EH1-EH7). The EH6 haplotype had the highest EA activity, significantly greater than the other haplotypes (*P* < 0.05). EH4 had the lowest EA activity, significantly lower than the other haplotypes except for EH5 (*P* < 0.05). **b** TAX content contrasts in different haplotypes (TH1-TH3). TH3 had the highest mean TAX content, and TH1 had the lowest mean TAX content, but neither reached significance level. The X-axis shows the haplotype of EA and TAX described in Fig. 4, and the Y-axis shows the corresponding EA activity and TAX content

**Fig. 7:**
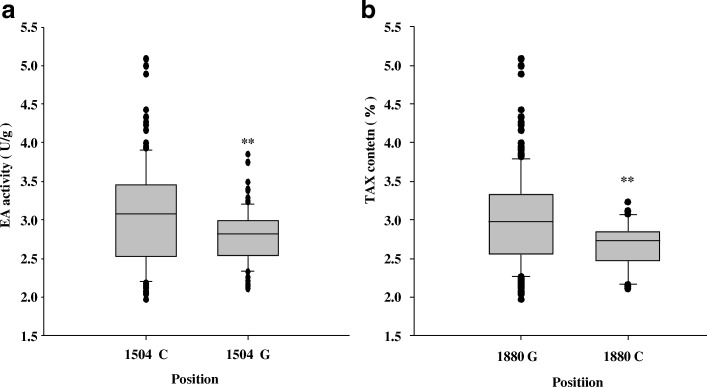
EA activity contrasts of different allelic forms. **a** At position 1504 bp, the EA activity of the C base form is significantly higher than that of the G base form. **b** At the position of 1880 bp, the EA activity of the G base form is significantly higher than that of the C form. The X-axis indicates the allele; the Y-axis shows the EA activity. ** indicates highly significant level (*P* < 0.01) of difference between means. –indicates that the base was deleted

Association analysis was performed to find possible links between nucleotide variation in *HvXYN1* and barley xylan-related traits. Sequencing identified 56 SNPs in the targeted region of *HvXYN1*. Among the 56 SNPs, 53 were from coding regions and three were from non-coding regions. Of the former, three were significantly associated with the two focal phenotypic traits. The SNPs at positions 110, 305, 1045, 1417, 1504, 1597, 1880 bp in the genomic region of *HvXYN1* were significantly associated with EA activity (*P* < 0. 001), and these explained 5.8, 5.8, 8.0, 8.8, 9.9, 6.3 and 7.0% of the phenotypic variation, respectively (Table 4, Additional file 2: Figure S1 and Additional file 3: Figure S3). Haplotype-trait associations were restricted to haplotypes with a higher than 5% frequency of minor alleles. The 210 barley accessions analyzed contained ten haplotypes (EH1-EH10) (Fig. 5a) according to the seven significant association SNPs (Table 4). Among these, the EH8-EH10 haplotypes were present in much lower frequency and were excluded from further analysis. Thus, only seven haplotypes (EH1-EH7) were used in the association analysis. The mean value of the EH6 haplotype for EA was 3.63 U/g; this was higher than those of haplotypes EH2 and EH3 (*P* < 0. 05) and was significantly higher than those of haplotypes EH1, EH4, and EH5 (*P* < 0. 01) (Fig. 6a and Additional file 4: Table S2). EH4 had the lowest endo-xylanase activity (2.644 U/g), significantly lower than the values of haplotypes EH1, EH2, EH3 and EH6 (*P* < 0.01). Further analysis revealed that the highest-activity (EH6) and lowest-activity (EH4) mutations were located at 1504 bp and 1880 bp, respectively, and that the activities of EH1 and EH2 were also significantly higher than that of EH3. The unique variation site was occurred at 1504 bp. This C-G transversion at 1504 bp may have caused the decrease in EA activity. The EA activity at 1504 bp allele C was 3.07 U/g, which was significantly higher than allele G (2.78 U/g) (Fig. 7a and Additional file 5: Table S3). Thus, SNP 1504 was considered as the candidate variant affecting EA activity. The variation of allele G to C at 1880 bp resulted in an amino acid mutation from valine to leucine. The phenotypic analysis revealed that EA activity at this site with G (3.01 U/g) was significantly higher than with C (2.66 U/g); we therefore speculated that the G/C mutation at 1880 bp resulted in the changes of EA activity. The sites 110, 305, and 1045 consisting of three haplotypes (TH1-TH3) (Fig. 5b, Additional file 6: Figure S2, and Additional file 3: Figure S3) were highly significantly associated with TAX content and explained 6.5, 6.5 and 5.4% of the phenotypic variation for TAX content, respectively. However, the TAX content of the three haplotypes did not reach significant differences. The highest (TH3) TAX content was 6.99% and the lowest (TH1) was 5.96%; the two values were not significantly different (*P* > 0.05) (Fig. 6b and Additional file 4: Table S2). Three SNPs (110, 305, 1045) significantly associated with EA activity and TAX content, which would be the key SNPs that affect the traits of xylan. There were seven SNPs significantly associated with EA activity, while only one marker in 1880 bp is biologically significant. Therefore, we speculate that 1880 bp can be used in marker-assisted selection to improve the quality of beer barley but it still need be further experiment validation.

**Table 4:** Significant associations between SNPs of *HvXYN1* and EA activity and TAX content of barley

Trait	Marker	Position	F	P	R^2^	nucleotide	amino acid
EA	1504	Exon	20.885	8.29E-06 **	0.099	C/G	G
EA	1417	Exon	18.675	2.39E-05**	0.088	A/G	V
EA	1045	Exon	16.908	5.62E-05**	0.080	C/T	N
EA	1597	Exon	13.454	3.09E-04**	0.063	A/G	A
EA	110	Intron	12.278	5.60E-04**	0.058	G/C	–
EA	305	Exon	12.278	5.60E-04**	0.058	G/A	P
EA	1880	Exon	7.374	8.03E-04**	0.070	G/−	V/L
TAX	110	Intron	13.750	2.68E-04**	0.065	G/C	–
TAX	305	Exon	13.750	2.68E-04**	0.065	G/A	P
TAX	1045	Exon	11.457	8.50E-04**	0.055	C/T	N

Note: The ‘Marker’ representing the nucleotide acid position in the genomic sequences of *HvXYN1*. R^2^ is the fraction of the total variation explained by the marker. * Indicates SNP significantly (*P* < 0.0001) associated with traits. ** Indicates SNP highly significantly (*P* < 0.00001) associated with traits

### Regional distribution of different haplotypes

**Fig. 8:**
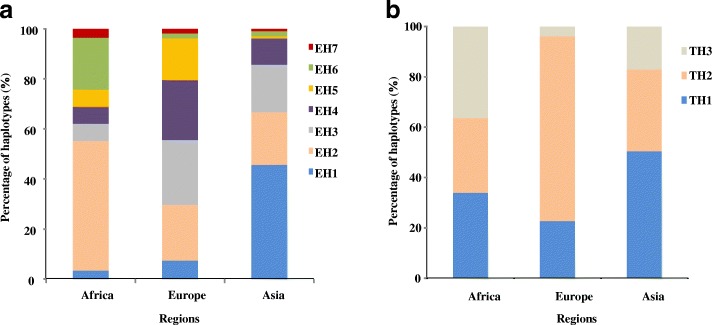
The geographic distribution of different haplotypes in *HvXYN1*. EH (EH1–EH7) and TH (TH1-TH3) represent haplotypes as described in Fig. 4. **a** The distribution of the haplotypes in EA activity among different geographic locations. **b** The distribution of the haplotypes in TAX content among different geographic location. The X axis represents the different geographic location, the Y axis represents percentage of haplotypes in EA activity and TAX content

Analyzing the distribution of haplotypes in different region, we found that the distributions of EH1, EH2, EH3, EH4, and EH5 were nonrandom. The haplotype EH6 was mainly distributed in Africa (Fig. 8a) and had higher enzyme activity (Fig. 6a). The lower-activity haplotype EH4 was mainly distributed in Europe and western Asia. This result was consistent with the EA active regional distribution, supporting the hypothesis that the EH6 haplotype is correlated with the high EA activity observed in Africa.

For the distribution of TAX traits, there were three haplotypes (TH1, TH2, and TH3) that were distributed in Africa, Europe, and Asia (Fig. 8b). TH1 had the most extensive distribution; TH2 was most widely distributed in Europe, followed by Africa and Asia; TH3 was less widely distributed in the three regions. The TH1 haplotype with the lowest TAX content was the Maximum distribution, while the TH3 haplotype with the highest TAX content had only three varieties, indicating that the TAX content in different regions was mainly affected by the TH1 haplotype. The genotype of the TH1 haplotype was consistent with the haplotypes EH1, EH2, EH3, EH4, and EH6 affecting EA activity. Moreover, EH4 had the lowest EA activity and EH6 had the highest, indicating that EA activity had little effect on TAX content. TH3 had three accessions, so the result is not representative.

## Discussion

The *XYN1* gene has been cloned in several grass plant species [22, 30, 31], but its key SNPs have not been characterized. In this study, 56 natural variation sites were identified from 1892 bp of the DNA sequence of *HvXYN1* across 210 barley accessions. The SNPs were distributed unevenly along the DNA sequence, with 53 and 3 variation sites in the exon and intron regions, respectively. Notably, the third exon contained the greatest nucleotide variation. Several studies have demonstrated a low level of polymorphism in exon regions and a high level in non-coding regions of barley [32–35], but the opposite result was observed in our research. There was a high level of polymorphism in exon regions of *HvXYN1*. It might be due to that the increased yield or disease resistance genes have gone through stronger selective pressures during domestication and breeding [36, 37]. *HvXYN1* encodes endo-beta-1,4-xylanase which mainly degraded xylan during germinating barley from the aleurone layer. Endo-beta-1,4-xylanase have an influence one content of xylan during seed germination, while it contributes little influences to the germination of seed or the growth of plants. The other reason is that, the domestication of barley, gene flow was not an isolated event but probably a continuous process. Indeed, the genome of a 6000-yr-old barley landrace carried signatures of the wild barley introgressions [38].

Tajima’s D measures the deviation from neutral evolution by comparing diversity estimates based on nucleotide diversity and average pairwise nucleotide diversity (π). In this study, the Tajima’s D test was performed on the *HvXYN1* sequence, yielding a value of − 1.44863 (Table 2). While this was below the significant statistically level, the values of D* and F* were significant, indicating that the evolution of the gene is basically in accord with the principle of neutral evolution. However, a negative Tajima’s D indicates population size expansion (for example, following a bottleneck or a selective sweep) or purifying selection [39–41]. This may be the reason for the low number of SNPs observed, in accord with previous research on other functional genes in barley [42–44]. However, in east Africa, the percent polymorphism was 0.0438, and the value of Tajima’s D was 2.123* and was statistically significant at the *P* < 0.05 level, suggesting that the gene may be experiencing negative selection to maintain a lower mutation rate, so that favorable alleles have been strongly positively selected [39–41]. Compared to previous research on barley [42, 45, 46], nucleotide diversity (π = 0.00736) and haplotype diversity (Hd = 0.923) of *HvXYN1* were relatively high in this study. The differences in SNP frequency among studies may be due to differences in genomic regions assayed, or the number, content and geographic origins of the germplasm used [47–49]. In comparing the gene polymorphisms of *HvXYN1* from different regions, the barley germplasm from East Asia showed the highest level of nucleic acid polymorphism (π = 0.0103), consistent with previous research results [44, 50, 51]. One probable reason is that these geographic regions were the main distribution areas of wild barley [52].

Association analysis is one powerful method to explore the relationship between sequence polymorphisms and phenotype variation [53﻿]. In the present study, seven variants of *HvXYN1* were significantly (*P* < 0.001) associated with xylan-related traits in barley. Among these, one variant at 110 bp of *HvXYN1* was within an intron region, while the other six at positions 305 bp, 1045 bp, 1417 bp, 1504 bp, 1597 bp and 1880 bp were within exons. Meanwhile, three variants (at positions 110 bp, 305 bp and 1045 bp) were associated with TAX content but were not significant. Xia et al. [44] discovered a synonymous mutation associated with phenotypic traits. In our study, there was also one SNP at 1504 bp, a synonymous mutation of G to C, that significantly increased EA activity (Fig. 7a), although this did not cause structural change of the gene product. A reasonable explanation for this phenomenon was the hitchhiking effect of a locus undergoing positive selection [54] or a false positive association. Thus, the SNPs identified by the association analysis should be validated before they can be applied to marker-assisted selection in the progeny [55, 56]. In addition, epigenetic or post-transcriptional regulation presumably affected the change in EA activity. Notably, a nonsynonymous amino acid substitution in the third exon of the *HvXYN1* gene at 1880 bp (C) has contributed to the low EA activity phenotype (Fig. 7b). Thus, the changes in the functional properties of the enzyme were likely caused by the change of bases [20, 57]. How the amino acid substitutions of different sequences impact the EA activity remains unclear. Further research using a quantitative xylanase method will be required to understand the relationship between non-synonymous mutations and EA activity. The change of V to L at this site may also indicate that it was an important SNP and that it can provide an important genetic resource for barley breeding research.

In agreement with the results of previous studies [58], TAX content had a greater CV (36%) in barley malt. One possible reason is that TAX content may be greatly affected by environmental factors. EA activity and TAX content were significantly and positively correlated (R^2^ = 0.135*) (Table 1) and the result suggest that EA activity and TAX content would be controlled by a common factor. We examined the geographical distribution of these haplotypes and found that there was no significant difference among the three haplotypes of TAX, but there was significantly higher diversity in Africa than in Europe and Asia. This may be due to the tropical location of Africa; for example, water deficiency and drought-induced stress could increase TAX content [59]. The EH6 haplotype with the highest EA activity (3.628 U/g) was mainly distributed in Africa, while the lowest (EH4) haplotype was mainly distributed in Europe. The current results suggested that AX content in malt could be not only related to EA but also be controlled by specific enzymes. Previous reports concerning the hydrolysis of AX during malting found that endoxylanase activity rose sharply after 72 h germination [60]. Thus, it is not difficult to understand why the accumulation of TAX content in African varieties induced enhanced EA activity in our study. In addition, barley has different uses in different regions. In European regions, barley is mostly used for beer brewing, which requires a lower TAX content; however, in Africa, barley is still an important food crop. Therefore, the EH6 haplotype that leads to high EA activity is mainly distributed in Africa, while the H4 haplotype with low EA activity is mainly found in Europe and Asia.

## Conclusion

This study identified 10 unique haplotypes based on 56 variations in *HvXYN1* among 210 barley accessions collected from 34 countries. Seven SNPs and seven haplotypes were significantly (*P* < 0.001) associated with EA activity and TAX content in barley. These SNPs can be applied as DNA markers in breeding programs to improve the quality of barley for beer brewing after further validation.

## Methods

### Plant materials and planting

A set of 210 barley (*Hordeum vulgare* L.) accessions sourced from 34 countries in three adjacent geographic regions, including Asia, Africa and Europe, (Table 3 and Additional file 7: Table S4). The 210 barley accessions were planted in Hangzhou Normal University (N30°19′7.12′′ N, E 120°23′7.89′′ E) test field. The sowing date were mid-November in 2014 and 2015, respectively. Each cultivar was grown in a plot, consisting of three lines. The plots were arranged by a randomized block design with three replications. At maturity, the middle line of each plot was harvested. After dried in an oven, about 20 g seeds from each plot were ground with a sample mill (Tesite instrument company, Tianjin, China) to pass through a 0.5-mm sieve and stored at ambient temperature for further chemical assay.

### PCR amplification and sequencing

Genomic DNA was extracted from leaf of each accession using CTAB method [61]. The primers used for DNA amplification were designed using the primer 3 online tool (http://primer3.ut.ee/). Through the primer walking technique, three overlapping oligos were designed to amplify 1926 bp of the gene. Details of primers were given in Additional file 8: Table S5. The PCR reactions were completed as following: 25 μL of 10 × KOD buffer, 10 μL of 2 mM dNTPs, 2.5 μL of 10 μM forward primers, 2.5 μL of 10 μM reverse primer, 4 μL of genomic DNA and 5 μL of ddH_2_O for each sample. The PCR amplification programs were as follows: initial denaturation at 94 °C for 2 min, followed by 34 cycles of denaturation at 98 °C for 10 s, annealing at 56 °C for 30 s, and extension at 68 °C for 1.0 min followed by final extension at 72 °C for 10 min. The PCR products were analyzed by 5% agarose gel electrophoresis in 0.5 × TBE buffer. All the product was sent to Hangzhou Zhixiu Technology Co. Ltd. for sequencing. Then the SeqMan program in the Lasergene software was used for splicing.

### Determination of total arabinoxylan (TAX) content in barley

Determination of total arabinoxylan (TAX) content, referred to the methods described by previous studies [58, 62, 63] with slightly improvements: 0.1 g sample mixed with 4 mL H2SO4 (1 mol L-1) in 1.5 mL centrifuge tube, then extracted in boiled water for 10 min, and cooled to room temperature. Centrifuged at 6000 rmp·min-1 for 5 min, took 1 mL of supernatant into a 15 mL centrifuge tube, added an equal volume of H2O, then added 10 mL of reaction solution (110 mL glacial acetic acid, 2 mL conc. HCL, 1 mL 1.75 g (1 mL glucose, 5 mL of 10% phloroglucinol-ethanol solution) and mixed. After reacted for 25 min in boiling water, cooled rapidly to room temperature and stopped the reaction. Determine by dual wavelength method (552 nm and 510 nm) and the difference in absorbance of the reaction solution was calculated, the total arabinoxylan content in the sample was calculated according to the standard curve. Each measurement was done with three replications.

### Determination of EA activity

Sprouted barley samples were homogenized in a mortar and grind rapidly in ice-cold homogenisation buffer (50 mM sodium acetate pH 5.0 containing 250 mM NaCl, 0.1% (*w*/*v*) polyvinyl polypyrrolidine (PVPP) and 0.5% (*v*/v) protease inhibitor cocktail (Sigma product P9599) at 2.5 ml/g malt [64]. The addition of 250 mM NaCl and PVPP enhanced recovery of endoxylanase activity. After leaving for 20 min at 0 °C, insoluble material was removed by centrifugation. The supernatant homogenate was filtered through Whatman GF/C glass fibre filters. Prior to analysis by dinitrosalicylic acid (DNS), fractional precipitation of the soluble extracted overnight against 10 mM sodium acetate pH 5.0. The dialysates were applied to a cation exchange column (Resource-S, Amersham Pharmacia Biotech Ltd) pre-equilibrated in 10 mM sodium acetate pH 5.0 and eluted with a gradient of 0–500 mM NaCl in the same buffer over 25 min.

The activity of endoxylanase was determined using dinitrosalicylic acid (DNS) [65, 66] with the commercial xylose (Sigma) as a standard curve. The basic principle of the method is that xylanase could catalyze xylanase to produce reduced sugars such as xylose under certain conditions. Reducing sugar with DNS could produce chromogenic reaction. 1.0% birchwood 4-O-methyl glucuronoxylan (Roth 7500) in 0.05 M Na-citrate buffer, pH 5.3. 1.0 g of xylan fully dissolved in 80 ml buffer at 60 °C and heated to the boiling point. Then cooled with continued stirring, covered and mixt slowly overnight. The following day made up to 100 ml with buffer. Store at 4 °C for a maximum of 1 week or freeze aliquots of e.g. 25 ml at − 20 °C~ Mix well after thawing. First added 1.8 ml oat xylan as substrate solution and 200 μL enzyme diluted appropriately in citrate buffer to a 15 ml test tube. Incubated the mixture for 5 min, at 50 °C. Then added 3.0 ml DNS, mixed and removed the tube to the water bath. Boil for 5 min, then cool quickly in cold water. Measure the colour produced at 540 nm against the reagent blank. Corrected the absorbance for background colour in the enzyme blank (add 200 μL buffer) if necessary. Used the standard line, convert the corrected absorbance to enzyme activity units (1 μmol/min). Calculate the activity, which is mean the amount of enzyme to generate 1 μmol xylose in one minute.

### Population structure analysis

The barley germplasm collection was structured into geographical groups in which some individuals were possibly related, so background genetic effects needed to be controlled to avoid spurious associations. Incorporating structure components as covariates in association analysis helps to reduce the false associations [67]. All of the accessions were genotyped using 35 SSR markers assigned to seven chromosomes of barley (Additional file 9: Table S6). Amplification of the SSRs was carried out as described by Hayden et al. [68]. Amplified PCR products were scored by comparing sizes between PCR products and a molecular weight ladder. Data from the 35 SSR markers were used to determine the population structure. Program STRUCTURE version 2.3.3 [69, 70] was used for population structure analysis, in which the number of clusters (k) was set from 2 to 11, and ten iterations were performed in an admixture model with a 10,000 iteration burn-in period and 100,000 MCMC (Markov Chain Monte Carlo) iterations. The most probable number of clusters (k) was estimated according to the value of ΔK. When ΔK had the highest value, the value of k indicated the number of clusters [71]. The optimal number of subpopulations was determined by the statistic ΔK based on the rate of change of the likelihood value [72].

### Association analysis and statistical analysis

The multiple genomic sequences were aligned by ClustalW 2.0.9 [73]. Sequence start and end adjustment was performed using the BioEdit V7.2.5 software, and the alignment results were used as input into TASSEL 3.0 [74], where the SNP was considered as a fixed effect. Association analysis between SNP markers in *XYN1* and EA activity and TAX content related traits was evaluated using a general linear model (GLM_Q) in TASSEL v3.0 (http://www.maizegenetics.net/tassel), where the SNP was considered as a fixed effect and the factor matrix of subpopulation membership (Q matrix) was used as a cofactor to account for population structure. The significance of associations between markers and traits was tested using an F-test. The association between a marker and a trait is represented by its R^2^ value, an estimate of the percentage of phenotypic variation explained by the marker. Haplotypes with a frequency < 5% were discarded to avoid biased associations. Nucleotide diversity (π), haplotype diversity, Tajima’s D [41] and D* and F* [39] values were calculated using the DnaSP 5.0 program [75]. Linkage disequilibrium (LD) was estimated by using standardized disequilibrium coefficients (D’) and squared allele-frequency correlations (R^2^) for pairs of SNP loci according to the TASSEL program. Polymorphism information content (PIC) was calculated as described in Kota et al. [76]. TASSEL was also used to identify SNP trait associations by generating a general linear model (GLM). The Duncan multiple range test and critical test were conducted if the analyses were significant (*P* < 0.05). Correlations between three traits and gene expression levels were examined by the Spearman correlation coefficient test. Statistical analysis was performed using the SPSS software. Phylogenetic analyses were conducted using the MEGA7 software with the following parameters: tested neighbor joining tree, Poisson correction, pairwise deletion, and bootstrap analysis with 1000 replicates [77].

## Additional files

Additional file 1: **Table S1.** EA activity and TAX content contrast in different regions. (DOCX 15 kb)

Additional file 2: **Figure S1.** Identification of EA by association analyses. Manhattan plots in the 210 accessions. and seven SNPs (red points) about *HvXYN1* identified in this study with EA activity. The X axis represents the physical position of *HvXYN1*, and Negative log10-transformed *P* values are plotted on the vertical axis. (PDF 81 kb)

Additional file 3: **Figure S3.** Identification of EA activity and TAX content by association analyses. Quantile-quantile (Q-Q) plots in the 210 accessions. Red points present EA activity and blue points present TAX content. The X axis represents the expected P values and P values are plotted on the vertical axis. (PDF 67 kb)

Additional file 4: **Table S2.** The mean value comparison of EA activity and TAX content in different haplotype. (DOCX 16 kb)

Additional file 5: **Table S3.** EA activity and TAX content contrast in different superior alleles. (DOCX 15 kb)

Additional file 6: **Figure S2.** Identification of TAX content by association analyses. Manhattan plots in the 210 accessions. And three SNPs (red points) about *HvXYN1* identified in this study with TAX content. The X axis represents the physical position of *HvXYN1*, and Negative log10-transformed P values are plotted on the vertical axis. (PDF 76 kb)

Additional file 7: **Table S4.** 210 barley accessions from 34 countries. (XLSX 13 kb)

Additional file 8: **Table S5.** List of overlapping primers used for the amplificati1on of 1.9 kb nucleotide region of *HvXYN1* gene. (DOCX 14 kb)

Additional file 9: **Table S6.** 35 SSR markers on 7 chromosomes. (XLSX 12 kb)

## Abbreviations

EA: endo-β-1,4-xylanase
SNPs: single nucleotide polymorphisms
TAX: total arabinoxylan
XYN1: Endo-β-1,4-xylanase1
