# Supplementary material for: Genetic variation of HvXYN1 associated with endoxylanase activity and TAX content in barley (Hordeum vulgare L.)
Source: BMC Plant Biol. 2019 Apr 30;19:170. doi: 10.1186/s12870-019-1747-5 (PMC6492322; doi:10.1186/s12870-019-1747-5)
Supplement: Supplementary file 1 — Table S1. EA activity and TAX content contrast in different regions. (DOCX 15 kb) [file 12870_2019_1747_MOESM1_ESM.docx]

**Table S1 EA activity and TAX content contrast in different regions**

|  |  | Mean | | Africa | Europe | Asia |
| --- | --- | --- | --- | --- | --- | --- |
| EA activity ( U/g ) | Africa | 3.487±0.577 |  | | 6.424** | 5.413** |
|  | Europe | 2.782±0.395 |  | |  | 0.772 |
|  | Asia | 2.71±0.793 |  | |  |  |
| TAX content ( %) | Africa | 7.985±2.84 |  | | 3.389** | 5.415** |
|  | Europe | 6.101±5.305 |  | |  | 2.448* |
|  | Asia | 5.305±1.497 |  | |  |  |
