# Supplementary figures and images for: Genetic variation of HvXYN1 associated with endoxylanase activity and TAX content in barley (Hordeum vulgare L.)
Source: BMC Plant Biol. 2019 Apr 30;19:170. doi: 10.1186/s12870-019-1747-5 (PMC6492322; doi:10.1186/s12870-019-1747-5)

P-Values by position for EA activity

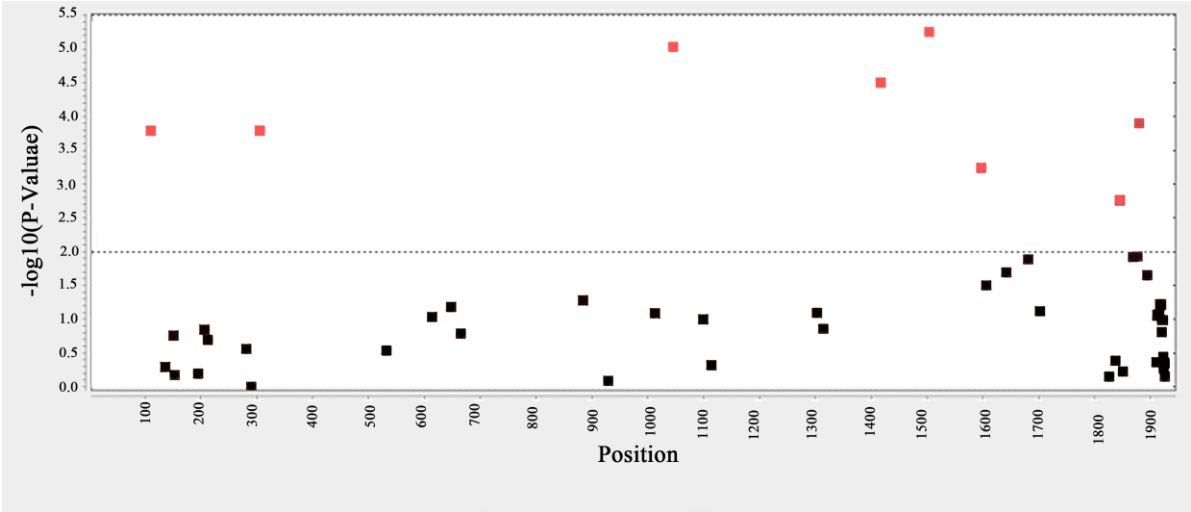

Figure S1

Supplement: Supplementary file 2 — Figure S1. Identification of EA by association analyses. Manhattan plots in the 210 accessions. and seven SNPs (red points) about HvXYN1 identified in this study with EA activity. The X axis represents the physical position of HvXYN1, and Negative log10-transformed P values are plotted on the vertical axis. (PDF 81 kb) [file 12870_2019_1747_MOESM2_ESM.pdf]

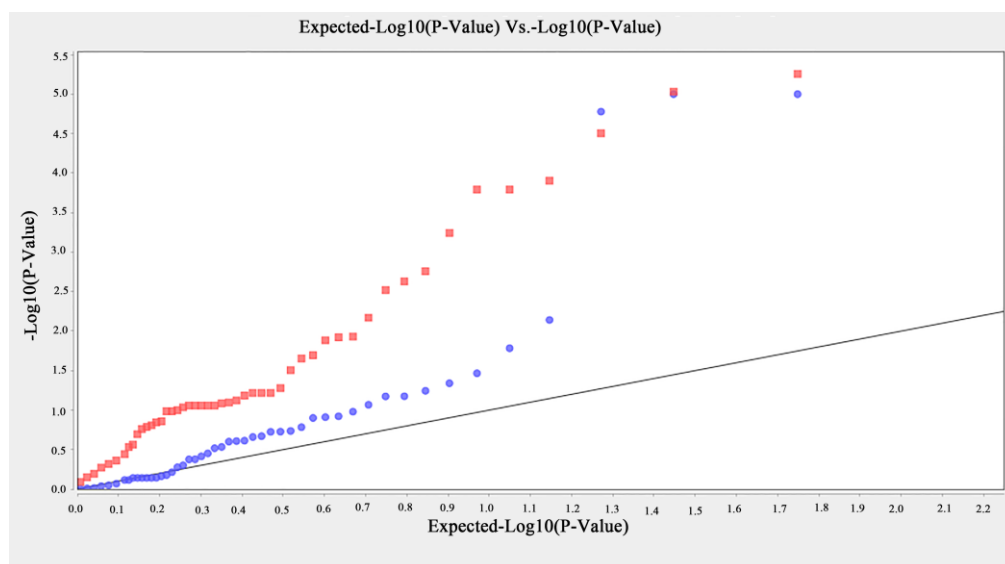

Figure S3

Supplement: Supplementary file 3 — Figure S3. Identification of EA activity and TAX content by association analyses. Quantile-quantile (Q-Q) plots in the 210 accessions. Red points present EA activity and blue points present TAX content. The X axis represents the expected P values and P values are plotted on the vertical axis. (PDF 67 kb) [file 12870_2019_1747_MOESM3_ESM.pdf]

P-Values by Position for TAX content

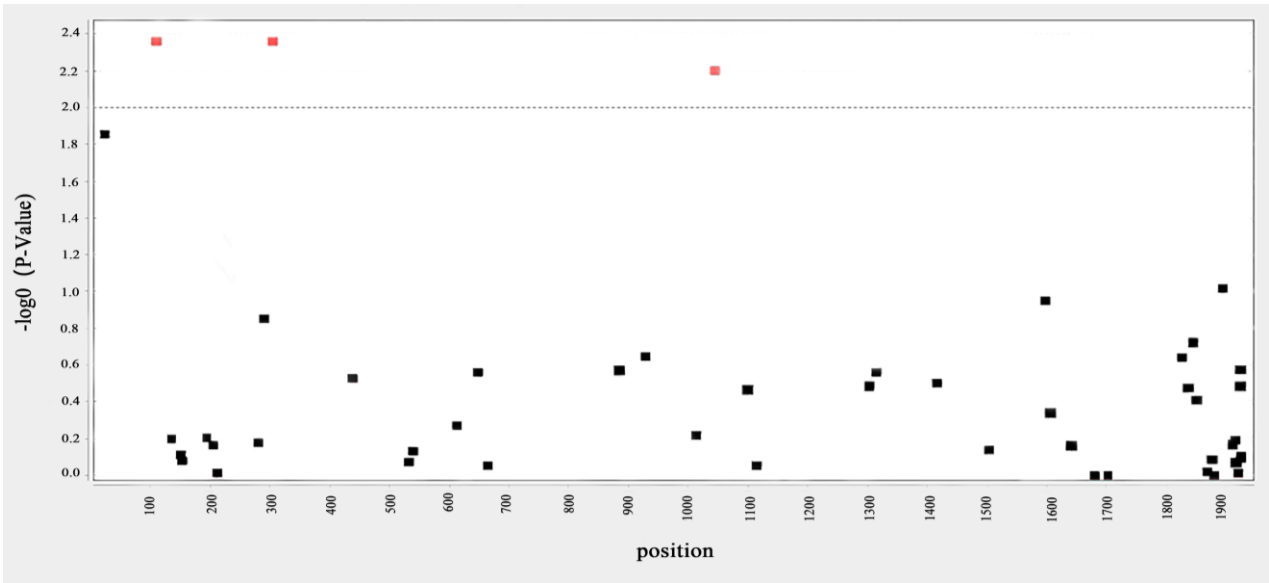

Figure S2

Supplement: Supplementary file 6 — Figure S2. Identification of TAX content by association analyses. Manhattan plots in the 210 accessions. And three SNPs (red points) about HvXYN1 identified in this study with TAX content. The X axis represents the physical position of HvXYN1, and Negative log10-transformed P values are plotted on the vertical axis. (PDF 76 kb) [file 12870_2019_1747_MOESM6_ESM.pdf]
