# Supplementary material for: Genetic variation of HvXYN1 associated with endoxylanase activity and TAX content in barley (Hordeum vulgare L.)
Source: BMC Plant Biol. 2019 Apr 30;19:170. doi: 10.1186/s12870-019-1747-5 (PMC6492322; doi:10.1186/s12870-019-1747-5)
Supplement: Supplementary file 4 — Table S2. The mean value comparison of EA activity and TAX content in different haplotype. (DOCX 16 kb) [file 12870_2019_1747_MOESM4_ESM.docx]

**Table S2 The mean vale comparison of EA activity and TAX content in different haplotype.**

| EA | N | Mean±SD | EH1 | EH2 | EH3 | EH4 | EH5 | EH6 | EH7 |
| --- | --- | --- | --- | --- | --- | --- | --- | --- | --- |
| EH1 | 60 | 2.98±0.57 |  | -0.97 | 1.10 | 3.77** | 1.40* | -3.04** | 2.31 |
| EH2 | 54 | 3.10±0.68 |  |  | 1.96 | 4.24** | 3.30* | -2.14* | 0.72 |
| EH3 | 38 | 2.88±0.37 |  |  |  | 3.50** | 1.05 | -3.01* | 0.47 |
| EH4 | 29 | 2.64±0.28 |  |  |  |  | 0.28 | -3.99** | -0.99 |
| EH5 | 12 | 2.76±0.37 |  |  |  |  |  | -3.34** | -2.32 |
| EH6 | 9 | 3.63±0.72 |  |  |  |  |  |  | 2.90 |
| EH7 | 3 | 2.81±0.23 |  |  |  |  |  |  |  |

Note: N, number of cultivars. tested. SD, standard deviation. * indicates significant level (P < 0.01) of difference between means. ** indicates highly significant level (P < 0.01) of difference between means.
