# Supplementary material for: Genetic variation of HvXYN1 associated with endoxylanase activity and TAX content in barley (Hordeum vulgare L.)
Source: BMC Plant Biol. 2019 Apr 30;19:170. doi: 10.1186/s12870-019-1747-5 (PMC6492322; doi:10.1186/s12870-019-1747-5)
Supplement: Supplementary file 5 — Table S3. EA activity and TAX content contrast in different superior alleles. (DOCX 15 kb) [file 12870_2019_1747_MOESM5_ESM.docx]

**Table S3 EA activity and TAX content contrast in different superior alleles.**

|  | Base | Mean | *P* | T |
| --- | --- | --- | --- | --- |
| EA 1504 | C | 3.077 |  | 4.088 |
|  | G | 2.792 | 0.00** |  |
| EA 1880 | G | 3.015 |  | 4.98 |
|  | C | 2.664 | 0.00** |  |

Note : ** indicates highly significant level (P < 0.01) of difference between means
