# Supplementary material for: Genetic variation of HvXYN1 associated with endoxylanase activity and TAX content in barley (Hordeum vulgare L.)
Source: BMC Plant Biol. 2019 Apr 30;19:170. doi: 10.1186/s12870-019-1747-5 (PMC6492322; doi:10.1186/s12870-019-1747-5)
Supplement: Supplementary file 8 — Table S5. List of overlapping primers used for the amplificati1on of 1.9 kb nucleotide region of HvXYN1 gene. (DOCX 14 kb) [file 12870_2019_1747_MOESM8_ESM.docx]

**Table S5 List of overlapping primers used for the ampliﬁcati1on of 1.9kb nucleotide region of *HvXYN1* gene.**

| Fragments | Forward Primer | Reverse Primer | Size (bp) |
| --- | --- | --- | --- |
| xy1 | tcacacagcagagatcatca | tcaccttgtcagtcttgtcc | 719 |
| xy2 | cgtcaaggtcatggatctc | tgtagtcgttgacgaagagc | 732 |
| xy3 | actacgacgtcaacaacgag | atcacttgacgtgttgcatg | 770 |
